# Species composition and insecticide resistance in malaria vectors in Ellibou, southern Côte d’Ivoire and first finding of *Anopheles arabiensis* in Côte d’Ivoire

**DOI:** 10.1186/s12936-023-04456-y

**Published:** 2023-03-13

**Authors:** Bédjou P. N’Dri, Nadja C. Wipf, Jasmina Saric, Behi K. Fodjo, Giovanna Raso, Jürg Utzinger, Pie Müller, Chouaïbou S. Mouhamadou

**Affiliations:** 1 Centre Suisse de Recherches Scientifiques en Côte d’Ivoire, 01 BP 1303 Abidjan 01, Côte d’Ivoire; 2 Swiss Tropical and Public Health Institute, Kreuzstrasse 2, 4123 Allschwil, Switzerland; 3 University of Basel, 4001 Basel, Switzerland; 4 North Carolina State University, Raleigh, NC 27695-7508 USA

**Keywords:** *Anopheles arabiensis*, *Anopheles gambiae*, Côte d’Ivoire, Insecticide resistance, Metabolic resistance, Malaria

## Abstract

**Background:**

Knowing the species composition and insecticide resistance status of the target vector population is important to guide malaria vector control. The aim of this study was to characterize the malaria vector population in terms of species composition, insecticide susceptibility status and potential underlying resistance mechanisms in Ellibou, southern Côte d’Ivoire.

**Methods:**

A 1-year longitudinal entomological survey was conducted using light traps and pyrethroid spray catches to sample adult mosquitoes in combination with larval sampling. The susceptibility status of *Anopheles gambiae sensu lato* (s.l.) to bendiocarb, deltamethrin, DDT and malathion was assessed using the World Health Organization insecticide susceptibility test. Additionally, *An. gambiae* specimens were screened for knockdown (*kdr*) and acetylcholineesterase (*ace1*) target site resistance alleles, and the expression levels of eight metabolic resistance genes, including seven cytochrome P450 monooxygenases (P450s) and one glutathione *S*-transferase (GST), measured with reverse transcription quantitative real-time polymerase chain reaction (qPCR).

**Results:**

Overall, 2383 adult mosquitoes from 12 different taxa were collected with *Culex quinquefasciatus* and *An. gambiae* being the predominant taxa. Molecular identification of *An. gambiae *s.l. revealed the presence of *Anopheles arabiensis*, *Anopheles coluzzii*, *An. gambiae sensu stricto* (s.s.) and *Anopheles coluzzii/An. gambiae *s.s. hybrids. *Anopheles gambiae* mosquitoes were resistant to all insecticides except malathion. PCR diagnostics revealed the presence of *ace1*-G280S and the *kdr* L995F, L995S and N1570Y target-site mutations. Additionally, several genes were upregulated, including five P450s (i.e., *CYP6P3*, *CYP6M2*, *CYP9K1*, *CYP6Z1*, *CYP6P1*) and *GSTE2.*

**Conclusion:**

This is the first documented presence of *An. arabiensis* in Côte d’Ivoire. Its detection – together with a recent finding further north of the country – confirms its existence in the country, which is an early warning sign, as *An. arabiensis* shows a different biology than the currently documented malaria vectors. Because the local *An. gambiae* population was still susceptible to malathion, upregulation of P450s, conferring insecticide resistance to pyrethroids, together with the presence of *ace1*, suggest negative cross-resistance. Therefore, organophosphates could be an alternative insecticide class for indoor residual spraying in the Ellibou area, while additional tools against the outdoor biting *An. arabiensis* will have to be considered.

**Supplementary Information:**

The online version contains supplementary material available at 10.1186/s12936-023-04456-y.

## Background

Malaria is a major public health problem in sub-Saharan Africa. In Côte d’Ivoire, malaria is the leading cause of mortality and hospitalization in children under the age of 5 years [1]. Across sub-Saharan Africa, the key malaria vectors are *Anopheles gambiae sensu stricto* (s.s.), *Anopheles coluzzii*, *Anopheles arabiensis* and *Anopheles funestus* [2]. In Côte d’Ivoire, *An. gambiae *s.s., *An. coluzii*, *Anopheles nili* and *An. funestus* are known vectors driving malaria transmission [3] with *An. gambiae *s.s. and *An. coluzzii* being the key vectors [4]. Recently, the presence of *An. arabiensis*, another important malaria vector, has been reported from Bouaké in the central part of Côte d’Ivoire, an observation that might be linked to the urbanization process [5].

The control of malaria vectors primarily relies on indoor residual spraying (IRS) and long-lasting insecticidal nets (LLINs) [6] with pyrethroids being the main class of insecticides for bed nets. However, susceptibility to pyrethroids has generally declined in *Anopheles* mosquitoes [4], including in Côte d’Ivoire, where insecticide resistance is widespread [4], representing a threat to the success of existing malaria control and elimination programmes as well as for the control of other vector-borne diseases.

Several molecular mechanisms are involved in conferring insecticide resistance, while the key mechanisms are metabolic resistance and target-site insensitivity. The most studied target-site insensitivity loci in *An. gambiae sensu lato* (s.l.) are point mutations in the voltage-gated sodium channel, conferring cross-resistance to pyrethroids and dichlorodiphenyltrichloroethane (DDT), including the L995F (formerly known as L1014F or *kdr* ‘west’) and L995S (formerly known as L1014S or *kdr* ‘east’) at the same codon position [7, 8]. Both *kdr* point mutations are present in *An. gambiae* across most of Africa and may co-occur in the same individual [9–13]. While L995F has been first detected in Côte d’Ivoire [14] and is now widely present, the L995S *kdr* has only recently been detected in Côte d’Ivoire in a single hybrid mosquito carrying both *kdr* mutations [15]. An additional *kdr* mutation—N1570Y (formerly known as N1575Y), the so called “super *kdr*”—occurs with and intensifies the effect of the L995F-mediated pyrethroid resistance and has recently also been detected in southern Côte d’Ivoire [16, 17]. Another key point mutation is the acetylcholinesterase *ace1*-G280S (formerly known as G119S) mutation and is associated with resistance to carbamates and organophosphates [18, 19]. Both *kdr* and *ace1*-G280S mutation were present in central and northern Côte d’Ivoire [20–22]. In contrast to target-site insensitivity, metabolic resistance is linked to overexpression of specific enzymes that break down, export or sequester insecticides. The three major enzyme families associated with metabolic insecticide resistance are carboxylesterases (COE), glutathione *S*-transferases (GSTs) and cytochrome P450 monooxygenases (P450s) [23].

Knowing the species composition and insecticide resistance status of the target vector population is important to guide malaria vector control. While several studies were conducted on malaria transmission, vector population and insecticide resistance in Côte d’Ivoire, highlighting the impact of irrigated rice farming on malaria transmission [22, 24–27], the picture across the country is still very patchy. This motivated the study reported here, carried out in the southern part of Côte d’Ivoire in the village of Ellibou, situated in a forest and ground swamp area suitable for malaria vectors. The village belongs to the region of Agneby-Tiassa and is located along the main motorway connecting Abidjan with the capital Yamoussoukro. Along this route, mosquitoes may be displaced through motorized vehicles. Thus far, Ellibou has not been the subject of entomological surveys. Given the behavioural and ecological differences among malaria vectors and the spread of resistance to public health insecticides across the country, a deeper understanding of local species composition and an accurate and updated insecticide resistance profile are central to the success of vector control. Hence, the overarching aim of this study was to characterize the local malaria vector population in Ellibou in terms of species composition, sporozoite rates, insecticide susceptibility status and potential underlying resistance mechanisms.

## Methods

### Study site

**Fig. 1:**
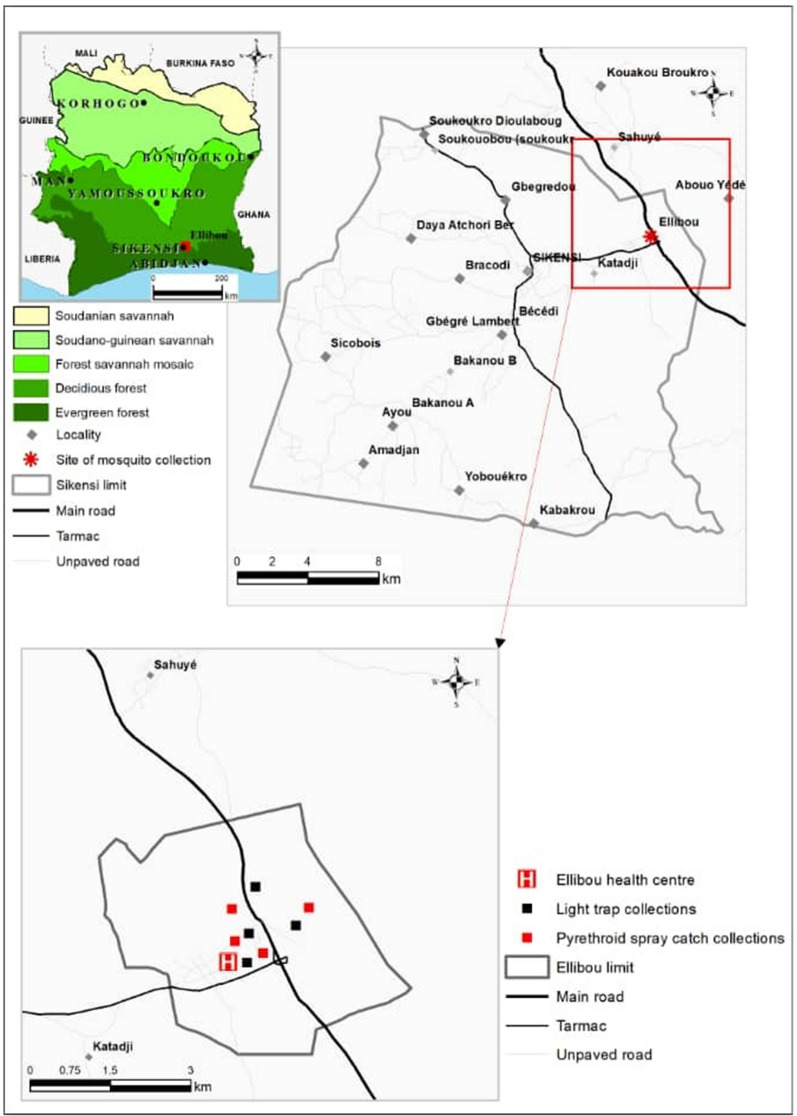
Map of Côte d’Ivoire showing the study site, Ellibou, where the mosquitoes were collected. The map was created using ArcGIS Desktop 10.7.1 (ESRI Inc., Redlands, CA, USA). Source of background map: CNTIG data (Comité National de Télédétection et d’Information Géographique), 2020

The study was carried out in Ellibou (geographical coordinates: 5°40ʹ59.4ʺ N latitude, 4°30ʹ31.1ʺ W longitude; Fig. 1) from January to December 2015. Ellibou has a population of approximately 12,000 inhabitants according to “Recensement Géneral de la Population et de l’Habitat 2021” and belongs administratively to the Agneby-Tiassa region. The village is located in a forested area, some 60 km north-west of Abidjan with direct access to a national motorway. Agriculture and trade are the main economic activities in Ellibou. The village is surrounded by uneven ground leading to many small temporary ponds that serve as breeding sites for mosquitoes during the rainy season. At the time of the study, insecticide-treated nets (ITNs) were the only vector control intervention implemented in Ellibou.

### Meteorological data

Meteorological data, including monthly average temperature and precipitation, were obtained from the local weather station of the Meteorological Department of the Society for Airport, Aeronautical and Meteorological Operations and Development in Côte d’Ivoire. The climate of Ellibou is characterized by four seasons: (i) a long rainy season from April to July; (ii) a short dry season in August and September; (iii) a short rainy season in October and November; and (iv) a long dry season from December to March.

### Mosquito sampling and morphological identification

Mosquitoes were sampled as either adults or larvae. Adult mosquitoes were collected from houses using Centers for Disease Control and Prevention (CDC) light traps and pyrethroid spray catches (PSC) twice per week from January to December 2015. Both collection techniques were performed on the same days. In addition, the number of people that slept in the rooms the previous night was recorded.

The CDC light traps (John W. Hock Company; Gainesville, FL, USA) were placed in four arbitrarily chosen houses across four sectors given by the main road and the bridge, dividing Ellibou into four sectors (Fig. 1). In each sector, two houses were selected, one house for PSC and another one for CDC light traps with a distance of approximately 100 m between the houses. In each house, a CDC light trap was placed inside one bed room. Inside the bed room, the trap was mounted at 1.5 m above the floor and close to the bed of the study participants that slept under a long-lasting insecticidal net (LLIN). While all other lights in the room were switched off, mosquitoes that entered the room were attracted by the incandescent light of the trap and then aspirated into a collection bag by the trap’s fan. The traps ran for two consecutive days in each week from 20:00 to 06:00 h. Each morning, the mosquitoes caught in the traps were removed and transferred to the laboratory at Centre Suisse de Recherches Scientifiques en Côte d’Ivoire (CSRS) in Abidjan for morphological identification. The mosquito species and sex, as well as the gonotrophic stage of the females (i.e. unfed, fed, semi-gravid or gravid) were recorded.

PSC were performed on two consecutive days per week in the mornings from 05:30 to 07:30 h in four additional, arbitrarily chosen houses by spraying a commercially available product “ORO” (Químicas ORO SA; San Antonio de Benagéber, Spain) containing pyrethrin and the synergist piperonyl butoxyde. Before spraying, a white sheet was placed on the floor, then the room was sprayed and any mosquitoes knocked down within 10–15 min after spraying were collected with forceps, put in Petri dishes and transferred to the entomology lab at CSRS for morphological identification.

For the insecticide susceptibility assays, the mosquitoes were collected as larvae during the long rainy season in June and July 2015. The larvae were sampled using a standardized dipper from breeding sites such as puddles on unpaved roads and around household courtyards within the village. Larvae that were morphologically identified as *Anopheles* sp. were transferred to the insectary at the CSRS and reared to adult stage. The larvae were fed ground Tetra MikroMin fish food (Tetra; Melle, Germany), while the emerging adult mosquitoes were provided with 10% honey solution *ad libitum*. All mosquito rearing was performed under controlled ambient environmental conditions with a temperature of 27 ± 2 °C, a relative humidity of 80 ± 4% and a 12:12 h light: dark photoperiod.

### Sporozoite rate

In addition to species identification and insecticide resistance testing, the female *Anopheles* caught inside the houses with PSCs were assessed for their gonotrophic status and for the presence of *Plasmodium falciparum* sporozoites. For the presence of sporozoites, the mosquitoes were screened using the circumsporozoite protein-based enzyme-linked immunosorbent assay (ELISA) from heads and thoraces from all PSC-sampled *Anopheles* mosquitoes following the protocol by Wirtz et al. [28].

### Insecticide susceptibility tests

The World Health Organization (WHO) insecticide susceptibility assays [29] were performed to assess the susceptibility of 3- to 5-day-old adult *An. gambiae* females reared from larval collections to diagnostic concentrations of deltamethrin (0.05%), DDT (4%), malathion (5%) and bendiocarb (0.1%). These insecticides were chosen because they represent the conventional insecticide classes for adult mosquitoes. The test papers were purchased from the official WHO provider, University Sains Malaysia.

In the experiments, batches of 20–25 mosquitoes per tube with four replicates (i.e. 80–100 mosquitoes) were exposed to each insecticide for 60 min and the number of specimens knocked down was recorded. Subsequently, the mosquitoes were transferred into holding tubes and supplied with 10% honey solution. After 24 h, the number of dead mosquitoes was recorded and insecticide resistance classification was performed based on WHO criteria [29]. The insecticide susceptible *An. gambiae *s.s. Kisumu strain was included in all assays as a control for the assay and for assessing the quality of the insecticide-impregnated test papers.

### Nucleic acids extraction

Non-blood fed adult *An. gambiae* females, emerging from the larval collections that had not been exposed to insecticides, were stored in RNA*later* (Ambion, Inc.; Austin, TX, USA). Nucleic acids were extracted from a subset of 141 specimens using MagaZorb^®^ DNA Mini-Prep kits (Promega Corporation; Madison, WI, USA) with slight modifications of the manufacturer’s protocol. The MagaZorb^®^ kit extracts both DNA and RNA simultaneously. In addition, nucleic acids were extracted from 2- to 3-day-old females of Kisumu (*n* = 50) and Ngousso strains (*n* = 50) and served as insecticide susceptible references.

Mosquitoes were ground on ice in 200 µl TE buffer using a plastic pestle in a 1.5 ml Eppendorf tube. Subsequently, 200 µl of lysis buffer was added and vortexed for 15 s. During a 10 min incubation period at room temperature (25 °C), the sample was repeatedly vortexed for 15 s every 2 min. To pellet the mosquito debris, the lysed sample was centrifuged for 2 min at 16,000 rcf and the clear supernatant was transferred into a 1.5 ml Eppendorf tube. Twenty microlitres of magnetic beads (MagaZorb^®^ Reagent) and 500 µl of binding buffer were added to the supernatant and vortexed for 15 s. The sample was again incubated for 10 min at room temperature and repeatedly vortexed. After letting the magnetic beads sediment settle by placing the tube on a magnetic rack for 2 min, the supernatant was discarded. Subsequently, the magnetic beads were washed two times as follows: 200 µl of wash buffer was added, then the mixture was vortexed and incubated at room temperature for 1 min. After sedimentation of the beads on a magnetic rack for 2 min, the supernatant was discarded. Finally, 180 µl elution buffer was added, the mixture was vortexed and incubated for 10 min at 50 °C while repeatedly vortexing. The sample was vortexed again, spun down and placed on a magnetic rack for 2 min. On ice, a new 1.5 ml tube was prepared in which the supernatant containing the purified DNA and RNA was collected. The nucleic acid extracts were stored at − 80 °C until subjecting them to a series of quantitative PCR (qPCR) assays.

### Molecular species identification

All qPCR reactions, including the species identification, target-site and metabolic resistance assays, were performed on a Bio-Rad CFX 96 real-time PCR machine (Bio-Rad; Hercules, CA, USA). In each reaction, 1 µl of the nucleic acids extract from individual mosquitoes was added to the one-step reverse transcription qPCR (RT-qPCR) master mix supplied by Fast Track Diagnostics (Esch-sur-Alzette, Luxembourg) together with the primers and probes at the concentrations described by Wipf et al. [30] to reach a total reaction volume of 10 µl. The following thermal cycle conditions were used for all assays: 50 °C for 15 min, 95 °C for 3 min, followed by 40 amplification cycles of 95 °C for 3 s and 60 °C for 30 s. The purified nucleic acids were subjected to two complementary TaqMan qPCR assays to identify the 141 specimens to species level within the *An. gambiae* complex with previously described adaptations to the original protocols put forth by Wipf et al. [30].

The first assay differentiated *An. coluzzii/An. gambiae *s.s. (Ag+) as a group from *An. arabiensis* (Aa+) and *Anopheles bwambae/Anopheles melas/Anopheles merus/Anopheles quadirannulatus* (Aq+) as a group [31]. The second assay distinguishes between *An. gambiae *s.s. (former molecular S-form) and *An. coluzzii* (former molecular M-form) in Ag + samples based on a short interspersed nuclear element (SINE) using common primers published by Santolamazza et al. [32] and probes by Wipf et al. [30].

For confirmation and in view of no prior records of *An. arabiensis* from Côte d’Ivoire at the time of this study in 2015, amplicons from a subset of 10 qPCR assays that identified *An. arabiensis* were repeated and their amplicons sequenced. The amplicons were sent to Microsynth AG (Balgach, Switzerland) for Sanger sequencing and searched using BLAST [33].

### Insecticide target-site mutation assays

Previously published qPCR protocols were followed to identify the presence of insecticide resistance alleles, including the *kdr* mutations N1570Y [17], L995F and L995S [34, 35] and the *ace1* mutation G280S [35].

### Gene expression levels of metabolic resistance loci

The expression levels of eight detox genes previously associated with metabolic resistance in *An. gambiae* were measured in nucleic acids extracted from RNA*later*-preserved, individual specimens that were raised from field-collected larvae. These genes included seven P450s (i.e. *CYP4G16*, *CYP9K1*, *CYP6M2*, *CYP6P1*, *CYP6P3*, *CYP6P4* and *CYP6Z1*) and one GST (*GSTE2*). The expression levels were measured by RT-qPCR Taqman triplex assays, developed by Mavridis et al. [36], in which the transcripts of the ribosomal protein S7 (*RPS7*) serves as an internal reference to adjust for the overall expression level. Gene expression levels of field-collected specimens from Ellibou were calculated relative to two insecticide-susceptible laboratory strains of the same species. *Anopheles gambiae* s.s. from the field were compared to *An. gambiae *s.s. Kisumu, originating from Kisumu, Kenya, and *An. coluzzii* from the field were compared to *An. coluzzii* Ngousso, originating from Yaoundé, Cameroon. No gene expression data for *An. coluzzii*/*An. gambiae *s.s. hybrids and *An. arabiensis* were included in this study, because of the low number of field samples from Ellibou and because there was no susceptible laboratory reference stain of the same species available. Gene expression assays were ran for 50 individuals per species both from the field and laboratory reference strain, but only qPCR results that pass pre-set quality thresholds were included, e.g. individuals whose *RPS7* quantification cycle (Cq) values were above 27.10 have been excluded from the analysis.

### Statistical analysis

The monthly frequencies of mosquitoes collected from both CDC light traps and PSC collections were determined. Data were double entered into an Excel spreadsheet (Windows 10, Microsoft Corporation; Redmond, WA, USA).

The level of significance was set at *α* = 0.05. Pearson product-moment correlation tests were performed in order to assess linear associations between the entomological parameters and meteorological data. The sporozoite rate was determined by calculating the proportion of mosquito found to be positive to the circumsporozoite protein. The 95% confidence interval (CI) for frequency was calculated by using the formula: $\widehat{p}+/- z^{*} (\widehat{p}(1 - \widehat{p})/n)^{0.5}$, where *z* is 1.96. Data from the WHO insecticide susceptibility assays were interpreted according to WHO criteria [29] in the respective mosquito population. For all qPCR assays, the Cq values were determined from the measured amplification curves using the Bio-Rad CFX Maestro 1.0 software (Bio-Rad Laboratories; Hercules, CA, USA). For the target-site qPCR assays, the allele frequencies of each *kdr* and the *ace1* mutation were calculated by using the Hardy-Weinberg formula: $f\left( R \right)\, = \,\left( {2n.RR\, + \,n.RS} \right)/2N$ where *n* represents the number of mosquitoes of a given genotype and *N* the total number of mosquitoes analyzed. For the gene expression analysis, Cq values of the metabolic resistance genes were normalized against the Cq values of the ‘housekeeping’ gene *RPS7* to account for total gene expression in each individual. Then, using the method of Pfaffl et al. [37] implemented the REST 2009 software version 2.0.13, the expression levels of the resistant *An. gambiae *s.s. and *An. coluzzii* field population were calculated relative to the susceptible Kisumu and Ngousso laboratory colonies, respectively.

## Results

### Mosquito species composition

During the 1-year study, 2383 adult female mosquitoes from 10 taxa were collected by CDC light traps and PSC. Almost two-thirds of all mosquitoes were caught in the PSCs (Table 1). About half of the mosquitoes were morphologically identified as *Culex quinquefasciatus*, constituting the most abundant mosquito species in Ellibou, followed by the malaria vector species of the Gambiae complex. In addition to *An. gambiae *s.l., the malaria vectors *An. funestus *s.l. and *Anopheles pharoensis* were present. Among *Anopheles* sp., members of the Gambiae complex were by far the most predominant taxon with higher proportions in the PSCs. *Aedes, Culex* and *Mansonia* were the other culicine genera collected in Ellibou, representing 57.8% and 72.0% of the total mosquito population using CDC light traps and PSCs, respectively.

**Table 1:** Species and numbers of adult Culicidae sampled indoors from Ellibou, Côte d’Ivoire, in 2015

Subfamily	Species	Sampling method			
		Both methods	CDC light trap		PSC
*Anophelinae*	*Anopheles gambiae * s.l.	865	238		627
	*Anopheles pharoensis*	8	5		3
	*Anopheles funestus * s.l.	7	4		3
*Culicinae*	*Culex quinquefasciatus*	1265	471		794
	*Culex nebulosus*	106	79		27
	*Culex decens*	45	37		8
	*Mansonia africana*	41	19		22
	*Culex cinereus*	26	17		9
	*Mansonia uniformis*	11	9		2
	*Aedes aegypti*	9	5		4
	Total	2383	884		1499

*n*: number of specimens caught by both sampling methods

*PSC* pyrethroid spray catch

**Fig. 2:**
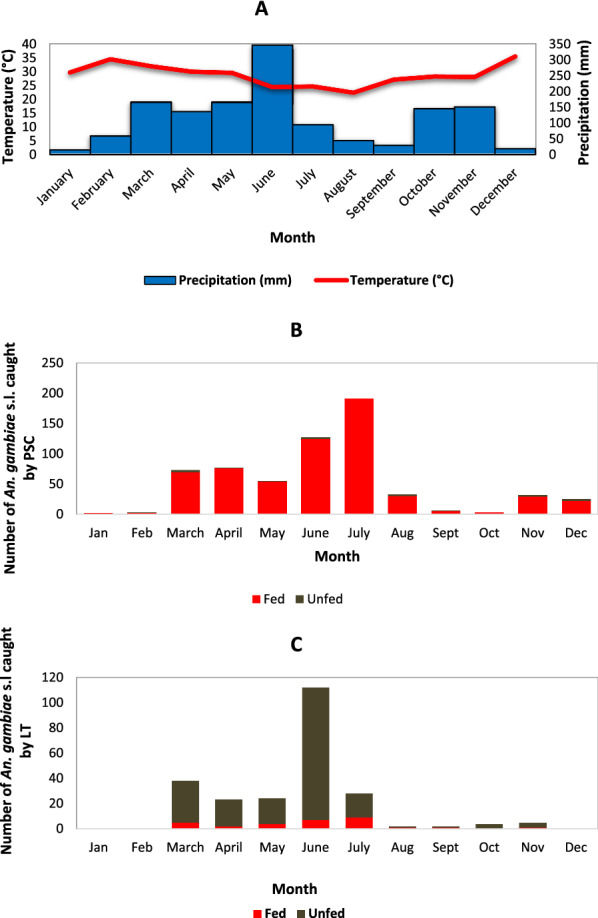
Seasonal weather conditions and changes in mosquito densities, Ellibou, Côte d’Ivoire, in 2015. Curves showing monthly variation in *An. gambiae *s.l. caught by PSC and CDC light trap: **A.** Monthly temperature and precipitation; **B. **Monthly caught fed and unfed *An. gambiae* s.l. by PSC; **C**. Monthly caught fed and unfed *An. gambiae *s.l. by CDC light trap

The numbers of adult *An. gambiae* mosquitoes collected inside the houses increased considerably when precipitation was above 200 mm per month with about a 1-month lag. The monthly average temperature in Ellibou was 26 °C, ranging between 20 and 30 °C and was not correlated with mosquito abundance (Fig. 2).

A total of 141 *An. gambiae *s.l. female adults reared from larvae collected in Ellibou were subjected to molecular species identification assays. For 13 individuals the qPCR reaction did not yield a signal; hence, these samples were excluded from further analysis. The molecular diagnostics revealed the presence of *An. arabiensis*, *An. coluzzii*, *An. gambiae *s.s. and hybrids between *An. coluzzii* and *An. gambiae *s.s.* Anopheles gambiae *s.s. (38.3%) and *An. coluzzii* (39.0%) were almost equally represented and 4.7% were *An. coluzzii/An. gambiae *s.s. hybrids. As the *An. arabiensis* finding is the first in Côte d’Ivoire at the time of the study, the amplicons of 10 specimens identified as *An. arabiensis* were sequenced for confirmation. All 10 sequences were identical with the *An. arabiensis* isolate NMNH2 ribosomal RNA intergenic spacer (GenBank: EU091308.1).

### Blood feeding and sporozoite rates

The number of blood-fed mosquitoes was linearly correlated with rainfall (*r* = 0.6, *p* < 0.05). Overall, 5.3% (95% CI 3.8–7.3%; *n* = 607) of tested blood-fed female *An. gambiae* were positive for sporozoites, while infective mosquitoes were detected throughout most of the year, except during the long dry season in January, February and December (Table 2).

**Table 2:** Seasonal variation in the number of blood-fed and proportion of sporozoite-positive *An. gambiae *s.l. in Ellibou, Côte d’Ivoire, in 2015

Season	Month	Blood-fed mosquitoes (*n*)	Sporozoite positive (%)^a^
Long dry	January	2	0.0 [0.0–65.8]
	February	2	0.0 [0.0–65.8]
	March	69	1.5 [0.1–7.8]
Long rainy	April	76	7.9 [3.7–16.2]
	May	54	9.3 [4.0–19.9]
	June	124	6.5 [3.3–12.2]
	July	191	0.5 [0.0–2.9]
Short dry	August	30	10.0 [3.5–25.6]
	September	5	100 [56.6–100]
Short rainy	October	3	66.7 [20.8–98.3]
	November	29	3.5 [0.2–17.2]
Long dry	December	22	0.0 [0.0–14.9]
Entire year		607	5.3 [3.8–7.3]

*n*: number of blood-fed *An. gambiae s.l.* mosquitoes caught using pyrethroid spray catches (PSC)

^a^ The number in brackets indicate the lower and upper limits of the 95% confidence intervals

### Insecticide susceptibility tests

To assess the insecticide resistance status of *An. gambiae* in Ellibou, adult females were raised from larvae collected from across Ellibou and then exposed to discriminating concentrations of the four conventional classes of insecticides (i.e. organochlorines, pyrethroids, organophosphates and carbamates).

A sample of 87 − 100 mosquitoes was exposed to an insecticide representing each insecticide class. In the positive controls, all *An. gambiae* Kisumu tested exhibited 98–100% mortality to all insecticides, indicating a good quality of the insecticide-impregnated papers, while exposure to control papers without insecticide resulted in mortality rates below 5%. The local *An. gambiae* population in Ellibou showed resistance to all insecticides tested, except for malathion (Table 3).

**Table 3:** Insecticide resistance status of *An. gambiae *s.l. from Ellibou, Côte d’Ivoire, in 2015

Colony	Insecticide	*n*	Knockdown (%)	Mortality (%)	Status
Ellibou	DDT 4%	94	0	0	Resistant
	Deltamethrin 0.05%	91	18	4	Resistant
	Bendiocarb 0.1%	87	69	52	Resistant
	Malathion 5%	87	60	98	Susceptible
Kisumu	DDT 4%	100	77	98	Susceptible
	Deltamethrin 0.05%	97	92	100	Susceptible
	Bendiocarb 0.1%	99	85	98	Susceptible
	Malathion 5%	97	85	100	Susceptible

*n*: total number of An. gambiae s.l. exposed in the WHO insecticide susceptibility test

### Molecular resistance mechanisms

All four target-site resistance markers for which mosquito samples were screened were present, including the *kdr* alleles L995S, L995F and N1570Y, and the *ace1*-G280S mutation. The L995F *kdr* mutation was predominant across *An. arabiensis*, *An. gambiae *s.s., *An. coluzzii* and *An. coluzzii/An. gambiae *s.s. hybrids with allelic frequencies ranging from 23 to 100% (Table 4). The highest rate was found in *An. gambiae *s.s. (100%). In contrast to L995F *kdr* mutation, the L995S mutant allele was only detected in *An. arabiensis*–at both homozygous and heterozygous state–with an allelic frequency of 65%. In addition, 7 out of 23 *An. arabiensis* individuals were found carrying the double 995 F and 995 S mutant alleles simultaneously. In contrast, the N1570Y *kdr* mutation was only found in *An. gambiae *s.s. albeit only at low frequency (8%). The *ace1*-G280S mutation was found in *An. arabiensis*, *An. coluzzii* and *An. gambiae *s.s. as well the *An. coluzzii*/*An. gambiae *s.s. hybrids with frequencies between 6% and 50% (Table 4 and Additional file 1: Table S4).

**Table 4:** *kdr* and *ace1* allele frequencies *in An. gambiae* s.l. from Ellibou, Côte d’Ivoire in 2015

Allele	Species	*n*	Genotype			Allele frequency (%)
			SS	RS	RR	
*kdr*-L995F	*An. arabiensis*	23	13	9*	1	23.9 [13.9–37.9]
	*An. coluzzii*	47	7	17	23	67.0 [57.0–75.7]
	*An. gambiae *s.s.	48	0	0	48	100 [92.6–100]
	*An. coluzzii*/*An. gambiae *s.s.	6	1	2	3	66.7 [22.2–95.7]
*kdr*-L995S	*An. arabiensis*	23	3	10*	10	65.2 [42.7–83.6]
	*An. coluzzii*	47	47	0	0	0 [0.0–7.5]
	*An. gambiae * s.s.	48	48	0	0	0 [0.0–7.4]
	*An. coluzzii*/*An. gambiae * s.s.	6	6	0	0	0 [0.0–45.9]
*kdr*-N1570Y	*An. arabiensis*	23	23	0	0	0 [0.0–14.8]
	*An. coluzzii*	47	47	0	0	0 [0.0–7.5]
	*An. gambiae * s.s.	48	41	6	1	8.3 [2.3–19.9]
	*An. coluzzii*/*An. gambiae * s.s.	6	6	0	0	0 [0.0–45.92]
*ace1*-G280S	*An. arabiensis*	23	20	3	0	6.5 [2.2–17.5]
	*An. coluzzii*	47	14	31	2	37.2 [28.1–47.3]
	*An. gambiae * s.s.	48	23	21	4	30.2 [21.9–40.0]
	*An. coluzzii*/*An. gambiae* s.s.	6	0	6	0	50 [11.8– 88.1]

*n*: total number of individuals screened

*RR* homozygous mutant; RS: heterozygous, *SS* homozygote wild type

^*^Seven individuals harboured the double 995 F and 995 S mutant alleles simultaneously

**Fig. 3:**
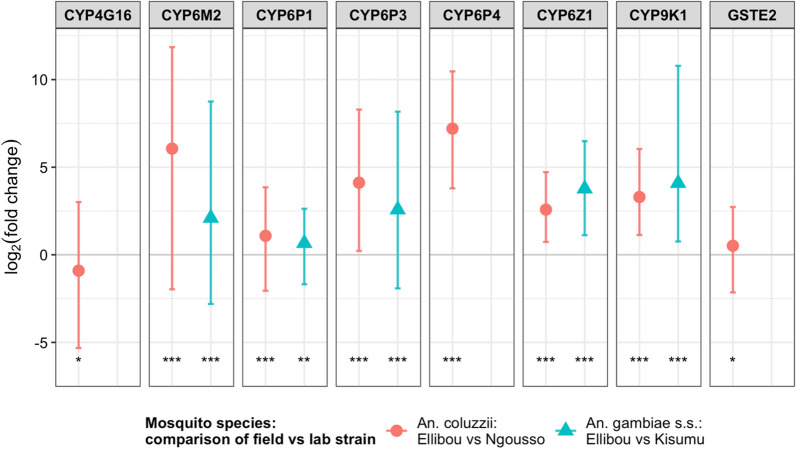
Expression levels of eight metabolic resistance genes in *An. coluzzii* (blue) and *An. gambiae* s.s. (red) from Ellibou compared to insecticide susceptible laboratory strains of the same species:* CYP4G16*, *CYP6M2*,* CYP6P1*, *CYP6P3, CYP6P4, CYP6Z1, CYP9K1 *and *GSTE2. * Genes with a log_2_ fold change above the thick, gray horizontal line (log_2_FC *> *0) were significantly higher expressed in the field population than in the reference lab populations, while genes below the horizontal line (log_2_FC *< *0) were significantly lower expressed in the field than in the lab population; Levels of significance: *p-value ≤ 0.05; **p-value  ≤ 0.01; and ***p-value ≤ 0.001, not plotted when p-value > 0.05

Expression levels of the measured metabolic resistance genes are shown in Fig. 3. All genes included in the expression analysis were significantly different expressed in *An. coluzzii* from Ellibou when compared to the susceptible Ngousso laboratory strain. The most upregulated genes were *CYP6P4* and CYP6M2, while *CYP4G16* was downregulated in the *An. coluzzii* field population. In *An. gambiae *s.s. from Ellibou less genes were expressed. The other genes *CYP6P4, CYP4G16* and *GSTE2* were statistically not significantly different expressed in the *An. gambiae *s.s*.* comparison (Additional file 2).

## Discussion

*Anopheles gambiae* was the principal malaria vectors identified in Ellibou, southern Côte d’Ivoire, with *P. falciparum*-infected individuals present throughout the year except for the long dry season. Species identification of larval collections showed that *An. coluzzii* and *An. gambiae *s.s. constituted the major malaria vectors at almost equal proportions, while 4.7% of the *An. gambiae *s.l. population were *An. coluzzii*/*An. gambiae *s.s. hybrids.

Surprisingly, 18% of the *An. gambiae* population were *An. arabiensis*, this being the first documented finding of *An. arabiensis* in Côte d’Ivoire at the time this study was conducted in 2015.

Recently, a study from Bouaké in the central part of Côte d’Ivoire also reported the presence of *An. arabiensis* [5] albeit the finding was 5 years following the one made here. Moreover, *An. arabiensis* is found in Côte d’Ivoire’s neighbouring countries, including Burkina Faso, Mali and Ghana [38–40] and across the West Africa region in countries, such as, Benin [41], Cabo Verde [42], Gambia [43], Guinea Bissau [44], Mauritania [45], Nigeria [46], Senegal [47] and Togo [48]. *Anopheles arabiensis* generally breeds and predominates in semi-arid and savannah areas [49, 50]. However, *An. arabiensis* may also breed in similar breeding sites to those of *An. gambiae* such as small temporary, sunlit and clear freshwater pools [50] and is found in more polluted breeding sites which might be linked to increased expression of detoxifying enzymes and target-site resistant loci [51].

In 2002, Ellibou experienced a transformation of its environment, explained by a sudden increase in population due to the socio-political crisis in Côte d’Ivoire, which could have indirectly led to more suitable habitats for *An. arabiensis*. Moreover, the motorway connecting Abidjan with the country’s capital, Yamoussoukro, crosses Ellibou village that constitutes a checkpoint for the control of all transport vehicles and their passengers. Therefore, it is conceivable that *An. arabiensis* was introduced to Ellibou by road traffic from neighbouring countries while the species was already pre-adapted to the altered local environment.

The presence of *An. arabiensis* in Côte d’Ivoire, now confirmed by two independent studies, is worrying and suggests that this major malaria vector is spreading further into new areas in West Africa. Indeed, in several West African cities, *An. arabiensis* has already become a dominant malaria vector [51–53]. In view of *An. arabiensis* primarily feeding outdoors [54], the effectiveness of IRS and LLINs might diminish should *An. arabiensis* become more dominant, requiring alternative intervention strategies. Worryingly, several point mutations conferring insecticide resistance were also identified in the *An. arabiensis* population of Ellibou, further challenging its control using insecticides.

The co-occurrence of *An. coluzzii* and *An. gambiae *s.s. is on par with other observations made across Côte d’Ivoire [55–57]. The presence of both sibling species at almost equal proportions, together with the presence of hybrids, suggests that Ellibou is an area were hybridization of the two sibling species takes place. In this respect it is conspicuous that both sibling species show a rather similar insecticide resistance profile which could be a result of hybridization. This is on par with the hypothesis that hybridization between the two species might have implications for the introgression of insecticide-resistance loci and wider consequences for malaria transmission [58].

A weakness of the current study is that, in contrast to the specimens reared from larval collections, the adult *An. gambiae* specimens were not further identified to sibling species level. Moreover, the collection methods used for the adult mosquitoes, including the CDC light traps and PSC, are biased towards mosquitoes biting and resting indoors. Therefore, the extent to which each sibling species and their hybrids contribute to malaria transmission in the Ellibou region—and Côte d’Ivoire as a whole—requires further investigations.

The *An. gambiae* population of Ellibou showed phenotypic resistance to deltamethrin, DDT and bendiocarb, yet was still susceptible to malathion. Parallel to phenotypic insecticide resistance, several target-site resistance alleles were identified, including the *kdr* L995F, L995S and N1570Y as well as the *ace1*-G280S mutation, although the *kdr* allele L995S was only present in *An. arabiensis*, where it was present in both homozygous and heterozygous state. In addition to the target-site resistance alleles, overexpression of P450s genes (mainly *CYP6M2, CYP6P3* and *CYP9K1*) confirm the observed pyrethroids resistance in the local malaria vectors. The phenotypic insecticide resistance data in the present study are similar to those reported from other settings in Côte d’Ivoire [4, 16, 30, 59] as is the high allelic frequency of the L995F *kdr* mutation in *An. gambiae *s.s. [59, 60]. The presence of the *kdr* N1570Y allele, in combination with the *kdr* L995F in *An. gambiae *s.s., may explain part of the resistance observed to deltamethrin in the Ellibou *An. gambiae *s.l. population [17]. With regards to *An. arabiensis* in Ellibou, similar results have been reported from Benin [61], Burkina Faso [62], Mauritania [45] and Senegal [63]. Another finding is that 7 *An. arabiensi*s specimens possessed both the L995F and L995S *kdr* simultaneously. This genotype has already been reported in *An. gambiae *s.s. from Uganda [64] and Côte d’Ivoire [59], but the present study seems to be the first record of an *An. arabiensis* L995F/L995S hybrid from Côte d’Ivoire. In addition to the *kdr* mutations, the overexpression of the five elevated P450 metabolic genes might also add to the pyrethroid and DDT cross-resistance in *An. gambiae *s.l. from Ellibou [65].

Intriguingly, despite the susceptibility of *An. gambiae *s.l. to malathion, *ace1-*G280S mutations conferring cross-resistance to carbamates and organophosphates were found across all three *An. gambiae* species from Ellibou. A possible explanation for this phenomenon is negative-cross resistance conferred by overexpression of P450s [30]. Indeed, several metabolic P450 enzymes, including *CYP6M2*, *CYP6P1, CYP6P3*, *CYP6Z1* and *CYP9K1*, were found to be upregulated and might have increased the toxicity of malathion [66, 67].

### Conclusion

The detection of *An. arabiensis* in southern Côte d’Ivoire, predating another finding further north of the country, confirms the presence of this malaria vector across the country. The presence of *An. arabiensis* is an early warning sign, as it shows a different behaviour and ecology than *An. coluzzii* and *An. gambiae *s.s., requiring different types of vector control interventions. It will be important to further investigate its spatial distribution, insecticide resistance status and contribution to malaria transmission for effective malaria control. Upregulation of P450s conferring insecticide resistance to pyrethroids together with the presence of *ace1* suggests negative cross-resistance since the local *An. gambiae *s.l. population was susceptible to malathion. Therefore, organophosphates could be an alternative insecticide class for IRS in the Ellibou area of southern Côte d’Ivoire.

## Supplementary Information

**Additional file 1.** Genotype of each individual mosquito of all analyzed insecticide resistance loci.

**Additional file 2: Table.** Genes expression analysis results between field strains and laboratory strains):*Anopheles gambiae* s.s. from the field were compared to *An. gambiae* s.s. Kisumu, originating fromKisumu, Kenya, and *An. coluzzii* from the field were compared to *An. coluzzii* Ngousso, originating fromYaoundé.

## Data Availability

Data and materials of this study are included in this article and its additional files.

## Abbreviations

*ace1*: acetylcholinesterase
CDC: Centers for Disease Control and Prevention
CI: Confidence interval
COE: Carboxylesterases
CSRS: Centre Suisse de Recherches Scientifiques en Côte d’Ivoire
DDT: Dichlorodiphenyltrichloro ethane
ELISA: Enzyme-linked immunosorbent assay
GST: Glutathione *S*-transferase
IRS: Indoor residual spraying
*kdr*: Knockdown resistance
LLIN: Long-lasting insecticidal net
PSC: Pyrethroid spray catch
RDT: Rapid diagnostic test
RT-qPCR: Reverse transcription quantitative polymerase chain reaction
SINE: Short interspersed nuclear element
SR: Sporozoite rate
WHO: World Health Organization
